# Self-care in people with long term health problems: a community based survey

**DOI:** 10.1186/1471-2296-12-53

**Published:** 2011-06-20

**Authors:** Fiona MacKichan, Charlotte Paterson, William E Henley, Nicky Britten

**Affiliations:** 1 Institite of Health Services Research, Peninsula Medical School, University of Exeter, Exeter EX2 4SG, UK

## Abstract

**Background:**

Self-care is a key component of current policies to manage long term conditions. Although most people with long-term health problems care for themselves within lay networks, consultation rates for long-term undifferentiated illness remain high. Promotion of self-care in these individuals requires an understanding of their own self-care practices and needs to be understood in the context of health care pluralism. The aim was to investigate the extent and nature of self-care practices in patients experiencing long term health problems, sources of information used for self-care, and use of other forms of health care (conventional health care and complementary and alternative medicine).

**Methods:**

The study involved a cross-sectional community-based survey set in three general practices in South West England: two in urban areas, one in a rural area. Data were collected using a postal questionnaire sent to a random sample of 3,060 registered adult patients. Respondents were asked to indicate which of six long term health problems they were experiencing, and to complete the questionnaire in reference to a single (most bothersome) problem only.

**Results:**

Of the 1,347 (45% unadjusted response rate) who responded, 583 reported having one or more of the six long term health problems and 572 completed the survey questionnaire. Use of self-care was notably more prevalent than other forms of health care. Nearly all respondents reported using self-care (mean of four self-care practices each). Predictors of high self-care reported in regression analysis included the reported number of health problems, bothersomeness of the health problem and having received a diagnosis. Although GPs were the most frequently used and trusted source of information, their advice was not associated with greater use of self-care.

**Conclusions:**

This study reveals both the high level and wide range of self-care practices undertaken by this population. It also highlights the importance of GPs as a source of trusted information and advice. Our findings suggest that in order to increase self-care without increasing consultation rates, GPs and other health care providers may need more resources to help them to endorse appropriate self-care practices and signpost patients to trusted sources of self-care support.

## Background

Kleinman's classic model of a health care system [1] portrays overlapping (and interacting) sectors of health care: the popular sector (including care provided by the individual person), professional (orthodox, or conventional, medical) and folk (e.g., complementary and alternative therapies). According to the model, whilst health care is plural, the popular sector is the largest component and is the nexus of health care; the place where decisions about health care--including consulting in the professional or folk sectors--are made. Individually based care, known as 'self-care', can be broadly defined as those behaviours that are practiced by the individual and directed at relieving symptoms, maintaining health, or preventing ill health [2]. Rogers and Hay [3] view self-care activity as a continuum, with those efforts made solely by the individual at one end and those shared with professionals at the other.

Research indicates that self-care for long term conditions requires many skills and resources; in a recent study, patients with diabetes were found to spend a mean of 58 minutes each day on specific self-care practices [4], and the Department of Health report that over three quarters of adults with long term conditions play an active role in caring for themselves 'all or most of the time' [5]. As such, self-care is an invaluable resource and constitutes an 'essential component' in the management of such conditions [6]. Concerns expressed 35 years ago that 'even a minor shift from self-care to doctor care could make intolerable demands on the general-practitioner service' [7] have continued to be voiced by GPs [6] and are now being taken a step further by NHS policies that promote self-care support as a potential mechanism for reducing the demand for medical care [8]. Such policies that support self-care are also considered to be a fundamental expression of patient-centeredness, and exemplify a health care service designed to meet the needs and preferences of patients.

The interactions between sectors of care, particularly the self-care/professional care (notably primary care) interface, are increasingly demanding attention in research. Studies have predominantly investigated self-management interventions relating to specific diseases or long term conditions, particularly diabetes, asthma and arthritis [9,10]. In contrast, those self-care practices adopted by people with less well-defined health problems are little understood, despite the prevalence of such problems and the challenges they pose to GPs [11,12]. For example, 20% of patients attending GPs and a third of patients attending neurology outpatients have unexplained physical symptoms [13,14]. Such problems bring to mind the important distinction between disease (as diagnosed pathology), and illness (as the subjective response to being unwell), which has been widely recognised [15]. In these 'swampy lowlands' of ongoing, complex physical and emotional health problems, the lack of effective medical interventions inevitably lends prominence to the promotion and support of self-care strategies.

Supporting self-care has been an inherent part of traditional general practice [16,17] and "an important but often hidden aspect of the supply of health care" [18]. However, there is a lack of research evidence relating to non-specific health problems, and subsequently in this study we were interested in health problems that might either be symptoms of a diagnosed condition, or constitute an undifferentiated health problem with no defined pathology. This, coupled with the fact that patients may be reluctant to disclose their use of self-care [19], leaves many GPs unaware of the extent to which such patients currently look after themselves and the types of self-care that they perceive as helpful. This is likely to limit the effectiveness of self-care support to these patients.

This study proposes to explore what self-care the public is already engaged with, taking a 'patient as provider' approach [20]; focusing on the end of the spectrum of self-care where efforts originating with the individual. We consider the place of self-care in the context of medical pluralism, and also ask: what day-to-day self-care practices are patients already using for themselves; what factors may be associated with greater use of self-care; and what sources of information for self-care do patients utilise, and value? This knowledge can inform self-care support at the consultation level, and can contribute to the development of self-care support initiatives that are responsive to patients' own agendas and self-care practices.

## Methods

### Design

The cross-sectional postal survey reported in this paper constituted the first phase of a mixed method study which aims to achieve an understanding of self-care behaviours in the community, focusing on common non-specific symptoms that are difficult to treat with medical interventions but which may be amenable to self-care practices. The purpose of the study is to enable hypothesis generation for further research and to inform the development of methods of supporting self-care in family practice.

### Setting and participants

The survey was conducted between January and March 2010 following approval from the South West Research Ethics Committee (reference 09/H0206/52). Three general practices in the South West of England were chosen to provide a study population with a range of socioeconomic and urban/rural characteristics. Furthermore, these practices could be seen to have varying expressed attitudes to self-care. The practices had a combined total list size of over 33,000 patients. A summary of the practices is provided (table 1).

**Table 1:** Participating GP practices

Practice	Location	No. full-time GPs	List size	Other information
Practice 1	Rural - Devon	10	13,691	A health centre with three separate surgeries. Expressed interest in self-care support and linked services (not funded through the NHS) include CAM

Practice 2	Urban - Bristol	11	11,570	A health centre with two surgeries. Located in a ward listed as amongst most deprived 20% in England (IMD 2007). No expressed interest in self-care support.

Practice 3	Urban - Exeter	7	8,503	A health centre with two surgeries. No expressed interest in self-care support.

At each practice, an electronic record search using Read codes produced a list of all patients aged over 18 excluding patients with severe and enduring mental illness, severe cognitive impairment and receiving palliative end-of-life care. The final list was stratified for age (18-39 yrs; 40-64 yrs; 65-101) and sex, and a random numbers table was used to draw a sample of 170 patients from each of the six age/sex bands, resulting in 1,020 patients per practice. The sampling strategy employed did not account for the consulting status of patients, meaning that those who responded may or may not have consulted their GP in relation to their long term health problem. The total study population of 3,060 was sent a survey questionnaire (additional file 1), information sheet and cover letter from their GP through the post. A reminder letter was sent to non-responders after two weeks. The questionnaire was also made available online for web-based submission (used by 24 respondents).

### Sample size

As the aims of the study were exploratory and the intention was not to provide definitive assessment of specific effects, an illustrative argument was used to justify the sample size. Under the assumption that at least 20% of primary care patients would have experienced the most common health problems during the past six months, and allowing for a 50% response rate, targeting a sample of 3,000 patients would result in recruitment of at least 100 patients for each condition. For a prevalence of use of self-care practices of at least 20% a sample size of this magnitude would make it possible to estimate condition-specific proportions of patients using the self-care practice with a margin of error of less than 9.8% at the 95% confidence level.

### Questionnaire

The survey questionnaire was designed specifically for this study and was structured around six health problems that were identified from the literature as being common long-term symptoms in the community and in primary care consultations [7,21,22], and as components of functional or medically unexplained conditions [10,12,23-25]. An initial symptom-based list from this literature was reduced to six items by comparing it with a list provided by participating GPs, in the context of including the illness groups of 'long term conditions', 'mental health', and 'women's health' identified in key Department of Health policy documents [26]. Our final list of health problems was: back pain, headaches or migraine, tiredness or fatigue, tummy or bowel problems, feeling stressed or anxious, and menstrual problems. The patient advisory group assisted in refining and describing the health problems to be investigated; the development and piloting of the survey questionnaire and the associated paperwork and procedures; and gave feedback on the presentation and discussion of the findings.

The questionnaire consisted of three sections: screening for the long term health problem(s), health care use and information seeking, and general health and demographics.

a) Screening for the long term health problem(s).These six health problems were presented on the first page of the questionnaire as a screening question: to be eligible for inclusion in the study patients need to have experienced one of the problems for the past six months, either all the time or intermittently. Patients could select more than one of the six problems, but were subsequently asked to complete the survey questionnaire in relation to the health problem that had been the most 'bothersome' to them (hereafter termed their primary problem). Those who checked the option 'none of these' (health problems) were directed to complete only the general health and demographic questions. The severity and impact of the primary health problem were measured on the five-item 'bothersomeness' scale [27], and respondents were asked whether they felt the health problem was under control.

b) Health care use and information seeking. The main part of the questionnaire focused on health care utilisation, including primary and secondary care, prescription medications and complementary and alternative therapies.

Self-care in the preceding six months were assessed using a 20-item scale (see appendix) of specific 'self provided' practices. Respondents were asked to report whether or not they had used each of the self-care practices in the preceding six months (the timescale allowed direct comparison with other forms of health care considered in the survey). The list of 20 practices were taken from an item pool drawn from a literature review of self-care behaviours for health problems such as chronic pain and fatigue [28-30] where approaches to self-care are less well defined that conditions such as diabetes. The full list was discussed and refined by the research team in consultation with the patients' group at one of the participating GP practices. Self-care activities were not categorised, instead post-hoc analysis is currently being undertaken to identify naturally occurring subgroups in these data.

The survey questionnaire additionally asked about use of, and trust in, a number of sources of information (e.g., pharmacist, web sites, family member). Two free text questions, 'is there anything else you think might help your health problem that you are not currently using?' and, 'is there anything you feel your GP could do to help with this health problem that is not already being done?' assessed unmet need.

c) General health and demographics. The final part of the questionnaire measured health status using the general health question from the SF-36, and collected demographic data (age, gender, household composition and ownership, education and ethnicity).

### Statistical analysis

Univariate analyses were performed to identify variables associated with use of a greater number of self-care practices. Correlation coefficients were interpreted according to the guidelines of Kraemer et al [31]: a correlation coefficient of 0.1 is said to be small or smaller than typical, 0.3 is said to be medium or typical, 0.5 is said to be large and 0.7 is said to be much larger than typical. Factors which showed significant associations at p < 0.1 were considered for entry into a multiple linear regression model. All multivariable models were first adjusted for age group and gender, the basis for stratifying the sample, together with primary health problem. Other variables considered for inclusion were classified into one of the following domains: (1) severity of primary health problem (including bothersomeness of problem, whether a diagnosis has been made and use of primary/secondary care); (2) patient complexity (the number of long-term health conditions and general state of health); (3) sources of health information used and (4) sources of health information trusted. The final multivariable model was developed by sequentially adding variables from each of these domains (in the order listed) using a forward stepwise method to select variables within each domain. The regression models were fitted using the R software [32] and all other analyses were conducted using SPSS 15.0 for windows.

## Results

**Figure 1:**
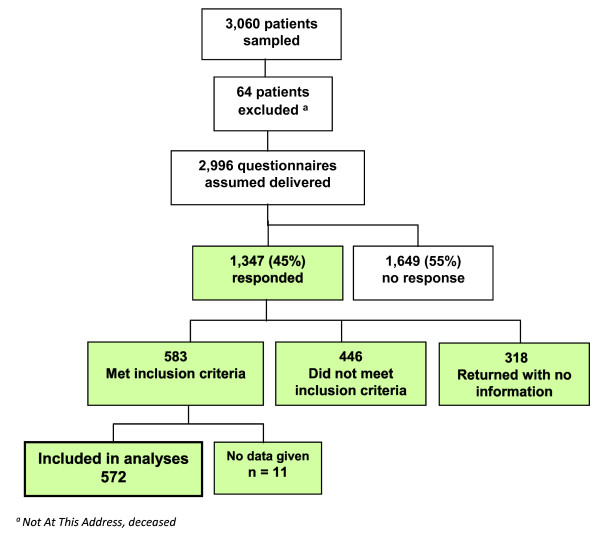
**Response flow chart**. A flow diagram illustrating the response to the postal survey.

A response rate flow chart is provided (Figure 1). Of the 3,060 questionnaires sent, 64 were returned as 'not at this address'; 1,347 (45%) patients responded. Of these, 583 reported having one of the six health problems and 572 of those provided data included in the analysis; 446 reported that they did not have one of the six long term health problems and the remaining 318 returned the questionnaire uncompleted.

### 1. Characteristics of the sample

The mean age was 57.9 (range 18-95), with 245 (43.2%) aged 65 and over, 218 (38.4%) aged 40-64 and 104 (18.3%) aged 18-39. Three hundred and fifty five (62.6%) of the sample were female and 538 (95.2%) gave their ethnicity as 'white'. Responders were more likely to be older and female than non-responders. Overall, 75.7% of the sample rated their general health as either 'excellent', 'very good' or 'good'; the remaining 24.3% rated it as 'fair' or 'poor'.

### 2. Health problems

Back pain was the most commonly reported health problem (n = 306, 53.5%), and was also most likely to be named as the primary (most bothersome) health problem (n = 200, 35%). It was common for respondents to have more than one of the index conditions; 63.8% reported having two or more of these health problems, and a third had three or more. Two hundred and twenty one respondents (38.6%) scored their primary health problem either 'extremely' or 'very' bothersome. The six health problems were reported as equally bothersome to respondents, with no statistically significant differences found. Overall, 192 (33.6%) of respondents indicated that they did not feel their health problem was under control.

### 3. Self-care and utilisation of other forms of health care

#### 3.1 Pluralism

**Figure 2:**
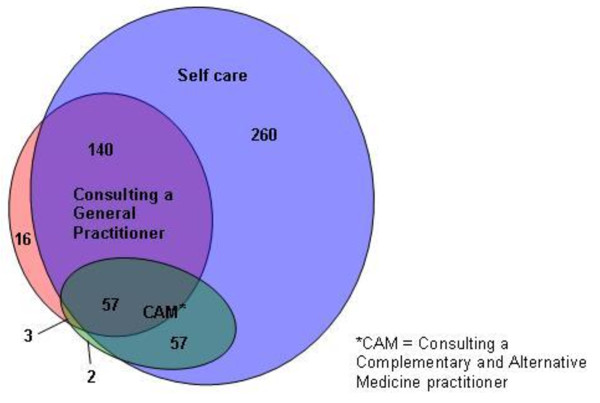
**Recent use of health care for six common long term health problems**. A Venn diagram illustrating the reported use of three different forms of health care (consulting a GP, consulting a CAM practitioner and use of self care) by survey respondents.

The Venn diagram (Figure 2) shows the relative use of self-care, primary health care (GP consulting), and complementary and alternative medicine (CAM) by the sample in the preceding six months. Thirty eight respondents reported no use of any health care. Although there was notable pluralism with overlap between the sectors of health care (and over one in ten of respondents reported recent use of all three forms), the diagram highlights the dominance of self provided care. Nearly half of respondents (48.6%) reported use of self-care alone. A further 26.2% reported use of self-care in combination with consulting a GP.

#### 3.2 Use of self-care

The use of self-care was widespread, with 90% of the sample (n = 515) reporting use of at least one of the 20 self-care practices listed in the survey (appendix A) in the past six months. The average number of self-care practices respondents reported using was four (range 0 - 14). Exercise and over the counter medications were the most popular forms of self-care, and were both reported by 43% of the sample. Both the number and type of self-care practices used varied according to the primary health problem, as shown in table 2. Respondents reporting their primary problem as back pain or feeling stressed or anxious used a greater number of self-care practices than those reporting other health problems.

**Table 2:** Numbers and percentages of respondents using specific self-care practices for their primary long term health problem, in whole sample and in each primary health problem subgroup

	Primary health problem (n)		Back Pain (200)	Headache/migraine (57)	Tiredness/fatigue (90)	Tummy/bowel (103)	Stress/anxiety (99)	Menstrual (23)
	Mean number of self-care practices reported		4.57	3.47	3.6	3.48	4.61	2.8

*Self-care practices*	*N*	*%*	*%*	*%*	*%*	*%*	*%*	*%*

Exercise	246	43	57	21.1	27.8	35.9	52.5	26.1
OTC medications	244	42.7	58.5	66.7	18.9	41.7	19.2	43.5
Positive thinking	211	36.9	29	19.3	38.9	36.9	63.6	26.1
Sleep	205	35.8	26.5	43.9	62.2	26.2	36.4	34.8
Heat	185	32.3	60	19.3	13.3	25.2	12.1	17.4
Pacing	160	28	35.5	15.8	33.3	11.7	35.4	13
Massage	145	25.3	47.5	28.1	8.9	13.6	10.1	8.7
Vitamins	132	23.1	24	19.3	28.9	25.2	14.1	30.4
Rest	131	22.9	22.5	33.3	30	16.5	18.2	21.7
Contact friend/family	111	19.4	10	8.8	13.3	13.6	56.6	17.4
Spend time alone	99	17.3	10.5	19.3	20	13.6	33.3	8.7
Reduce activity	80	14	17.5	8.8	16.7	9.7	14.1	4.3
Prayer	79	13.8	10.5	7	11.1	16.5	26.3	4.3
Diet	78	13.6	6.5	8.8	13.3	38.8	5.1	13
Meditation	67	11.7	12.5	7	6.7	13.6	17.2	4.3
Drinking	47	8.2	6.5	3.5	4.4	2.9	23.2	8.7
Cold	42	7.3	14.5	14	2.2	1	1	4.3
Hobbies	41	7.2	6.5	1.8	6.7	3.9	17.2	0
Support group	16	2.8	2	1.8	3.3	1.9	6.1	0

Univariate analysis suggested that a number of variables relating to general health, health care use and information seeking were associated with the use of a higher number of self-care practices in the whole sample (table 3 and table 4). Analysis of variables relating to socio-economic status (not shown) present a mixed picture, with home ownership being associated with less use of self care, and no association between level of self-care and school leaving age. Interestingly, those respondents registered with the participating GP practice which was located in an area of notable deprivation reported use of a greater number of self-care practices.

**Table 3:** Univariate analysis: Correlations between number of self-care practices used and numerical variables

Variable			Interpretation
	*r*	*p *	

Age	-0.180	< 0.01	Small inverse association between age and self-care; younger adults use a greater number of self-care practices
Number of health conditions	0.272	< 0.01	Typical positive association between the number of long term health problems (of the 6 listed) and number of self-care practices used
Bothersomeness	0.325	< 0.01	Bothersomeness of the primary health problem has a typical positive association with number of self-care practices used
General health	-0.142	< 0.01	Poorer self reported general health has a smaller than typical association with higher number of self-care practices
Number of sources of information utilised	0.482	< 0.01	Large positive association between the number of sources of information used and higher number of self-care practices
Number of sources of information trusted	0.387	< 0.01	Larger than typical positive association between the number of sources of information trusted and higher number of self-care practices

**Table 4:** Univariate analysis: Categorical factors associated with greater self-care

Variable	Self-care practices used Mean (SD)	p*	Interpretation
Sex		< 0.001	Female respondents used a greater number of self-care practices than male respondents
Male	3.12 (2.6)		
Female	4.62 (2.9)		
Problem under control		0.001	Those who felt their health problem was not under control used a greater number of self-care practices
Yes	3.75 (2.7)		
No	4.59 (2.9)		
Use of prescription medication		0.039	Those currently taking prescription medications used a greater number of self-care practices
Yes	4.36 (3.1)		
No	3.85 (2.7)		
Have consulted a GP (< 6 months)		< 0.001	Those who had consulted a GP recently used a greater number of self-care practices
Yes	4.8 (3.1)		
No	3.61 (2.6)		
Have consulted a CAM practitioner (< 6 months)		< 0.001	Those who had consulted a CAM practitioner used a greater number of self-care practices
Yes	5.64 (3)		
No	3.64 (2.7)		
Have seen a specialist (ever)		< 0.001	Those who had seen a specialist used a greater number of self-care practices
Yes	4.84 (3.0)		
No	3.66 (2.73)		

*p-value from two-sample t-test for equality of means

The multiple linear regression analysis (table 5) indicated that seven factors independently predicted higher self-care, after adjustment for age and sex. These were: the primary long-term health problem (with back pain being associated with the largest number of self-care practices), the bothersomeness of this problem, having a diagnosis, having seen a specialist, the number of long term problems experienced (of six), use of a variety of sources of information, and trust in formal sources of information. Age had an inverse relationship with self-care and men reported higher self-care than women. The final model accounted for 40% of variation in self-care scores (adjusted R^2^). Use of different sources of information was a particularly strong predictor, by itself accounting for 13.1% of the variance in self-care scores. The regression also suggests that different sources of information contributed in different ways to use of self-care; reported information seeking from nurses, pharmacists, CAM therapists, friends, family and newspapers/magazines had a notable influence on the number of self-care practices used.

**Table 5:** Multiple linear regression model: Determinants of greater self-care

Step	Variable	R^2^	R^2 ^change	Final β	95% CI
1	Age group	0.088	0.088		
	18 - 40			0.79*	0.20 to 1.38
	41 - 64			0.50*	0.02 to 0.97
	65+			0.00	
	Sex:				
	Male			0.96**	0.54 to 1.37
	Female			0.00	
2	Primary long-term health problem	0.135	0.047		
	Headaches or migraine			-1.12**	-1.82 to -0.42
	Tiredness or fatigue			-0.84*	-1.45 to -0.23
	Tummy or bowel problems			-1.40**	-1.97 to -0.82
	Feeling stressed or anxious			-0.62*	-1.23 to -0.02
	Menstrual problems			-2.87**	-3.92 to -1.83
	Back pain			0.00	
3	Bothersomeness of problem	0.217	0.082	0.47**	0.23 to 0.72
4	Having a diagnosis	0.243	0.026	0.54*	0.10 to 0.98
5	Have seen a specialist	0.252	0.009	0.49*	0.04 to 0.94
6	Number of long term health conditions	0.265	0.013	0.23*	0.05 to 0.40
7	Sources of health information	0.396	0.131		
	GP			0.01	-0.45 to 0.48
	Nurse			0.80*	0.21 to 1.39
	Pharmacist			0.71*	0.01 to 1.41
	Therapist			0.71	-0.03 to 1.44
	Complementary and alternative medicine			0.89*	0.23 to 1.55
	Friend			1.09**	0.52 to 1.66
	Family member			0.94**	0.44 to 1.45
	Internet chat room/forum			0.50	-1.12 to 2.12
	Support group			0.48	-0.93 to 1.89
	Newspaper or magazine			0.79*	0.03 to 1.54
	TV programme			0.62	-0.38 to 1.61
	Web sites			0.18	-0.34 to 0.71
8	Sources of information trusted	0.400	0.004		
	Formal (GP, nurse, pharmacist, therapist, CAM)			0.20*	0.04 to 0.36
	Informal (Friend, family, forum, support group)			0.06	-0.22 to 0.34
	Media (Paper, TV, website)			-0.10	-0.44 to 0.24

β = regression coefficient

* p < 0.05, ** p < 0.005

Regression coefficients (betas) for variables with mutually exclusive categories (including age, sex and presenting problem) are expressed relative to a reference category. For variables, such as sources of information used, where respondents can select more than one category, there is no reference category: regression coefficients indicate the mean number of additional self-care practices for respondents selecting a particular category compared to respondents not choosing that category

#### 3.3 Use of conventional health care

Overall, 77.4% (n = 443) reporting having consulted their GP about their health problem at some point, and 37.7% (n = 216) had consulted in the preceding six months. A third (n = 191, 33.4%) had seen a specialist and medication was taken by 41.4% (n = 235). There was variation in the use of conventional health care on the basis of differing primary health problems. Respondents with tummy or bowel problems were more likely to have recently used conventional health care when compared with the rest of the sample. This included being more likely to have consulted a GP (50.5%), χ^2 ^= 8.909, *p *= 0.01, odds ratio = 1.93, and used prescription medication (58.3%), χ^2 ^= 15.297, *p *< 0.001, odds ratio = 2.34, in the past six months. In comparison with the remaining sample as a whole, respondents with back pain were least likely to have consulted a GP in the preceding six months (29,.5%), χ^2 ^= 8.574, *p *= 0.01, odds ratio = 0.57, and those with tiredness and fatigue were least likely to report having received a diagnosis (29.5%), χ^2 ^= 8.574, *p *= 0.01, odds ratio = 0.45.

#### 3.4 use of Complementary and Alternative Medicine (CAM)

Overall, 118 respondents (20.6%) reported having consulted a CAM practitioner for their primary health problem over the preceding six months. Osteopathy or chiropractic was the most frequently reported form of CAM, used by 43 respondents--7.5% of the entire sample and 36.5% of those reporting use of CAM. In comparison with the remaining sample, those reporting back pain as their primary problem were notably more likely to have consulted a CAM practitioner (30%), χ^2 ^= 16.492, *p *< 0.001, odds ratio = 2.32.

### 4. Sources of Information

Overall, three quarters (n = 436) of respondents reported having sought out information about their health problem in the previous six months. The most popular source of information was the GP (n = 261, 45.6%), although family (n = 147, 25.7%), friends (n = 104, 18.2%) and websites (n = 125, 21.9%) were also widely reported. The use of sources of information did not necessarily reflect the extent to which respondents reported trust in them. More 'formal' sources of information (GP, nurse, pharmacist, CAM therapist) were highly trusted; 92.5% of the sample (n = 530) reported that they would trust one of these sources, compared with 'informal' sources such as friends and family and internet chat rooms (n = 241, 42.1%) and media sources, such as newspapers and television (n = 144, 25.1%). Sixteen percent of respondents (n = 92) reported that they would trust their GP alone as a source of information for their primary health problem.

## Discussion

Long standing health problems increasingly pose a challenge to health services. In the UK, service reform has focused on self-care and self management interventions both as a way of meeting the gap between demand and supply, and in parallel reflecting the preferences and priorities of service users, seeking to empower those experiencing long term conditions to 'take more control of their health'[33]. Complex and poorly defined health problems, whilst presenting unique dilemmas to primary care, have tended to invite less attention in research and the development of self-care interventions. In this context, it is important to achieve an understanding of what people are doing for themselves so that family practice can respond adequately to their needs and support self-care efforts.

Based on this study, use of self-care dominates the response to long term undifferentiated problems, although there was variation in the specific practices adopted depending on the health problem reported. Although our findings show that self-care was often used alone, medical pluralism was also evident and Figure 2 provides an updated version of Kleinman's classic diagram of a health care system [1]. The high use of self-care compared to seeking help from professional care suggests that there has been little change since the 'illness iceberg' was first demonstrated nearly 40 years ago [7,34]. However, it appears that the manner in which people care for themselves may be different, especially in the use of exercise. Our respondents indicated that exercise was used as commonly as 'over the counter medication' (both used by 43% of the sample), whereas a UK survey in 1976 did not feature exercise at all [7]. This may partly reflect methodological differences and cultural norms but it may also be that the evidence-based advice to people with back pain that exercise is beneficial is having an effect.

Some of our findings may be particularly relevant to health care providers. Firstly, the co-existence of several of the index conditions in the community--a third of respondents reported experiencing three or more of the six health problems-suggests that disease specific evidence for self-care interventions may be useful only in the context of discussions between patients and health care professionals that lead to personalised advice. In addition, factors such as timing and characteristics of the health problem are likely to be important in a patient's willingness and ability to engage with self-care support (see also [35]). Secondly, the finding that GPs were reported to be the most common and trusted sources of information about self-care poses a potential contradiction for GPs who are seeking to reduce consultations with patients with chronic, non-specific symptoms [8,36]. It appears that GPs, and other health care providers, may need to find sources of trusted self-care support and information which they can confidently recommend to their patients, although this poses its own challenges. Our survey also found that GPs did not have a notable influence on self-care practices, which may also indicate a need for further resources. Finally, we have the weaker, but still notable, association between increased self-care and self-report of having a diagnosis. This suggests that even in the absence of a medical diagnosis based on organic pathology, time spent sharing medical and lay explanations and the relative safety of possible self-care strategies may reap long-term rewards - a hypothesis that is supported by evidence about the importance of explanations in medically unexplained symptoms [37]. We are investigating these issues further in a nested qualitative study that will explore patients' expectations, needs and experiences of self-care support from GPs.

As an approach founded on self reliance and empowerment, with (arguably) the potential to impact on demand for services, support for self-care is often seen as an inherently 'good idea'. However, as has been discussed elsewhere [8,37], evidence does not necessarily support the idea of engendering self-care as a means of reducing demand. This study has illustrated the notable breadth and amount of self provided health care being undertaken by people with long term undifferentiated health problems, indicating that individuals already feel a sense of responsibility for managing their health problems. A further caveat is required; a persons ability and desire to engage with self-care is likely to be impacted (and limited) by a number of issues, and support for self-care demands 'a recognition...of the unequal resources that people have available to respond to and manage illness' [[38]:1819].

### Strengths and limitations

The cross-sectional design inevitably produces a snapshot of the phenomenon of self-care, which is unable to explore the changing use of self-care over the illness trajectory. We have explored this important issue in our linked qualitative research, to be published elsewhere, and additional longitudinal research would be valuable. Although a survey response rate of 45% is reasonable given that the population was a random sample of registered patients and eligibility for inclusion in the study was self reported, it constitutes one of the limitations of this study. However, as we might expect non-responders to contain a higher proportion of people without the eligible health problems, the response rate amongst eligible participants is likely to be higher than 45%.

Our findings can therefore be considered moderately generalisable across the population of the South West of England and probably beyond, although certain limitations exist. Our practice sample and patient stratification resulted in a study population that has an appropriate distribution of demographic and socioeconomic characteristics, however, 95% of respondents self-defined their ethnicity as 'white' (87.5% for England & Wales in 2001 census), which limits generalisability. A complicated picture emerged in relation to socio-economic status, and no conclusions can be drawn from this study; we recommend further investigation in this area, especially to ensure that self-care support initiatives address the varying contexts of patients. Although we also recruited the practices that were likely to have varying approaches to self-care support, the impact of the practice characteristics on patients' use of self-care is hard to delineate and may impact judgment of generalisability. However, we used a community sample, thereby not restricting responses to only those who have sought help in primary care (37.7% reported consulting a GP for their health problem in the preceding six months). An additional strength of the study is the involvement of patients in the design of the survey questionnaire.

Our findings on the frequent co-existence of several of the six index conditions would have been more meaningful if we had been able to collect data on other long-term health problems and chronic diseases. Understanding multi-morbidity and the interconnections between symptom complexes is a complex and important subject and highly relevant to self-care [39]. To do this within our study would have resulted in a longer and more complex questionnaire, but such an analysis would be important to pursue in further work.

A lack of recent UK community-based research into self-care practices for long term health problems meant that we carried out an exploratory study without any predefined hypotheses. As such, the findings will be a useful basis for further definitive studies in other populations. There are a number of areas where further research would be beneficial, including measuring the changing use of self-care over the illness trajectory and the ability of self-care practices to ameliorate symptoms. We are exploring these in a linked qualitative study involving adults experiencing long term back pain purposively sampled from survey respondents.

## Conclusions

The use of self-care, whether alone or in combination with other forms of health care, is the predominant approach to managing long term conditions. This study highlights how the composition and level of self-care (and nature of medical pluralism) may vary according to specific health problems. Despite the extent of self-care, the conventional health care sector can have a central role in supporting patients, given the extent to which health care providers are trusted sources of information and advice. Bringing discussion of self-care into the consultation, where appropriate, could help reinforce such practices. The ability to signpost is likely to be key, which itself may be dependent on the provision of resources that can be utilised by the practice team.

## Competing interests

The authors declare that they have no competing interests.

## Authors' contributions

NB and CP conceived and supervised the study. FM carried out the study. WH advised on and carried out some of the statistical analyses. FM and CP drafted the manuscript. All authors reviewed and approved the manuscript.

## Pre-publication history

The pre-publication history for this paper can be accessed here:

http://www.biomedcentral.com/1471-2296/12/53/prepub

## Supplementary Material

Additional file 1 **Self care survey questionnaire**. The study survey questionnaire document. Click here for file

## References

[B1] Kleinman A Patients and Healers in the Context of Culture 1980 Berkeley: University of California Press

[B2] Barofsky I Compliance, adherence and therapeutic alliance: Steps in the development of self care Soc Sci Med 1978 12 369 76 705382

[B3] Rogers AH S Self-Care and Access to Health Care. Opportunities for Improving Access to Care NHSE Anglia and Oxford Region 1998 Milton Keynes

[B4] Safford MM Russell L Suh DC Roman S Pogach L How much time do patients with diabetes spend on self-care? J Am Board Fam Pract 2005 18 4 262 70 10.3122/jabfm.18.4.262 15994472

[B5] Department of Health Self Care: A National View in 2007 Compared to 2004-05 2008 London: Crown Copyright

[B6] Jones R Self Care: Important for health services and needing more research BMJ 2000 320 596 10.1136/bmj.320.7235.596 10698860 PMC1117637

[B7] Morrell DC Wale CJ Symptoms perceived and recorded by patients J R Coll Gen Pract 1976 26 167 398 403 957305 PMC2157930

[B8] Chapple A Rogers A 'Self-care' and its relevance to developing demand management strategies: a review of qualitative research Health & Social Care in the Community 1999 7 6 445 54 10.1046/j.1365-2524.1999.00212.x 11560661

[B9] Barlow J Wright C Sheasby J Turner A Hainsworth J Self-management approaches for people with chronic conditions: a review Patient Education & Counseling 2002 48 2 177 87 10.1016/S0738-3991(02)00032-0 12401421

[B10] Newman S Steed L Mulligan K Self-management interventions for chronic illness Lancet 2004 364 9444 1523 37 10.1016/S0140-6736(04)17277-2 15500899

[B11] Aggarwal VR McBeth J Zakrzewska JM Lunt M Macfarlane GJ The epidemiology of chronic syndromes that are frequently unexplained: do they have common associated factors? Int J Epidemiol 2006 35 2 468 76 16303810 10.1093/ije/dyi265

[B12] Schon DS The reflective practitioner. How professional think in action 1995 Aldershot: Arena

[B13] Peveler R Kilkenny L Kinmonth AL Medically unexplained physical symptoms in primary care: A comparison of self report screening questionnaires and clinical opinion J Psychosom Res 1997 42 245 52 10.1016/S0022-3999(96)00292-9 9130181

[B14] Carson AJ Best S Postma K Stone J Warlow C Sharpe M The outcome of neurology outpatients with medically unexplained symptoms: a prospective cohort study J Neurol 2003 74 897 900 10.1136/jnnp.74.7.897 PMC1738573 12810775

[B15] Helman CG Disease versus illness in general practice Journal of the Royal College of General Practice 1981 31 548 52 PMC1972172 7328537

[B16] Balint M The Doctor, the Patient and His Illness 1957 London: Tavistock

[B17] Greaves CJ Campbell JL Supporting self-care in general practice Br J Gen Pract 2007 57 814 21 17925140 PMC2151815

[B18] Rogers A Entwistle V Pencheon D Managing demand - A patient led NHS: Managing demand at the interface between lay and primary care BMJ 1998 316 7147 1816 1819 9624078 10.1136/bmj.316.7147.1816 PMC1113323

[B19] Stevenson FA Britten N Barry CA Bradley CP Barber N Self-treatment and its discussion in medical consultations: how is medical pluralism managed in practice? Soc Sci Med 2003 57 3 513 27 10.1016/S0277-9536(02)00377-5 12791493

[B20] Segall A Goldstein J Exploring the Correlates of Self-Provided Health-Care Behavior Soc Sci Med 1989 29 2 153 61 10.1016/0277-9536(89)90163-9 2787533

[B21] Verbrugge LM Ascione FJ Exploring the Iceberg - Common Symptoms and How People Care for Them Med Care 1987 25 6 539 69 10.1097/00005650-198706000-00008 3695661

[B22] Kroenke K Price RK Symptoms in the Community - Prevalence, Classification, and Psychiatric Comorbidity Arch Intern Med 1993 153 21 2474 80 10.1001/archinte.153.21.2474 8215752

[B23] Mayou R Farmer A ABC of psychological medicine - Functional somatic symptoms and syndromes BMJ 2002 325 7358 265 8 10.1136/bmj.325.7358.265 12153926 PMC1123778

[B24] Henningsen P Zipfel S Herzog W Management of functional somatic syndromes Lancet 2007 369 9565 946 55 10.1016/S0140-6736(07)60159-7 17368156

[B25] Burton C Beyond somatisation: a review of the understanding and treatment of medically unexplained physical symptoms (MUPS) British Journal of General Practice 2003 53 488 231 9 14694702 PMC1314551

[B26] Department of Health Research evidence on the effectiveness of self-care support 2007 Crown Copyright

[B27] Dunn KM Croft PR Classification of low back pain in primary care: using "bothersomeness" to identify the most severe patients Spine 2005 30 1887 92 10.1097/01.brs.0000173900.46863.02 16103861

[B28] Blyth FM March LM Nicholas MK Cousins MJ Self-management of chronic pain: a population-based study Pain 2005 113 285 92 10.1016/j.pain.2004.12.004 15661435

[B29] Richardson A Ream EK Self-care behaviours initiated by chemotherapy patients in response to fatigue Int J Nurs Stud 1997 34 35 43 10.1016/S0020-7489(96)00031-4 9055119

[B30] Katz PP Use of self-management behaviors to cope with rheumatoid arthritis stressors Arthritis Rheum 2005 53 939 49 10.1002/art.21580 16342099

[B31] Kraemer HC Morgan GA Leech NL Gliner JA Vaske JJ Harmon RJ Measures of clinical significance J Am Acad Child Adolesc Psychiatry 2003 42 12 1524 9 10.1097/00004583-200312000-00022 14627890

[B32] R Development Core Team R A language and environment for statistical computing 2010 Vienna, Austria: R Foundation for Statistical Computing

[B33] Department of Health Improving chronic disease management 2004 The Stationery Office, London

[B34] Last JM The iceberg - Completing Clinical Picture in General Practice Lancet 1963 2 729 28 10.1093/ije/dyt113 24415602

[B35] Protheroe J Rogers A Kennedy AP Macdonald W Lee V Promoting patient engagement with self-management support information: a qualitative meta-synthesis of processes influencing uptake Implementation Science 2008 3 10.1186/1748-5908-3-44 PMC2575203 18851743

[B36] Townsend A Wyke S Hunt K Frequent consulting and multiple morbidity: a qualitative comparison of 'high' and 'low' consulters of GPs Fam Pract 2008 25 3 168 75 10.1093/fampra/cmn017 18448858 PMC2440493

[B37] Salmon P Peters S Stanley I Patients' perceptions of medical explanations for somatisation disorders: qualitative analysis BMJ 1999 318 7180 372 6 9933202 10.1136/bmj.318.7180.372 PMC27727

[B38] Rogers A Entwistle V Pencheon D A patient led NHS: managing demand at the interface between lay and primary care BMJ 1998 316 1816 9 9624078 10.1136/bmj.316.7147.1816 PMC1113323

[B39] Valderas JM Starfield B Sibbald B Salisbury C Roland M Defining comorbidity: implications for understanding health and health services Ann Fam Med 2009 7 4 357 63 10.1370/afm.983 19597174 PMC2713155

