# Supplementary material for: Self-care in people with long term health problems: a community based survey
Source: BMC Fam Pract. 2011 Jun 20;12:53. doi: 10.1186/1471-2296-12-53 (PMC3143929; doi:10.1186/1471-2296-12-53)
Supplement: Additional file 1 — Self care survey questionnaire. The study survey questionnaire document. [file 1471-2296-12-53-S1.PDF]

looking after ourselves

The Integrated Self Care in Family Practice Project

PENINSULA MEDICAL SCHOOL
UNIVERSITIES OF EXETER & PLYMOUTH

INSTRUCTIONS:

This questionnaire is about the things you do to help yourself with certain health problems. Please answer *every* question. Most questions can be answered simply by ticking the appropriate box ✓

Once you have finished, please return the questionnaire and the separate REPLY SLIP to us in the FREEPOST envelope provided. If you would prefer, you can fill the questionnaire in online at:

www.

All your answers will be treated as strictly confidential and only seen by the research team.

If you would like a copy of this questionnaire in large print, or have any difficulty with the questions, please call Fiona MacKichan on 01392 262913, or email her at: Fiona.mackichan@pms.ac.uk

Firstly, please look at the list below. Have you experienced any of the following **for the past 6 months or longer** (whether all the time or on and off)?

Please tick ✓ **all** that apply.

- Back pain ☐ a
- Headaches or migraine ☐ b
- Tiredness or fatigue ☐ c
- Tummy or bowel problems ☐ d
- Feeling stressed or anxious ☐ e
- Menstrual problems ☐ f

None of the above ☐ g — If you ticked this box (‘none of the above’), we are unable to include you in the study. We would be grateful if you would still **return this questionnaire** to us. Thank you.

ID label here

1. Please tell us which health problem has been the most bothersome to you **over the past 6 months**. Please tick ✓ **ONE**

Back pain	☐ 1	Please tick only one
Headaches or migraine	☐ 2	
Tiredness or fatigue	☐ 3	
Tummy or bowel problems	☐ 4	
Feeling stressed or anxious	☐ 5	
Menstrual problems	☐ 6	

Please answer the rest of the questionnaire thinking about this problem ONLY

2. Over the past 6 months, how much has this problem bothered you? Please tick only one

Not at all	Slightly	Moderately	Very much	Extremely
☐ 1	☐ 2	☐ 3	☐ 4	☐ 5

3. Do you feel that this health problem is under control? Yes ☐ 1 No ☐ 2

*Please **circle** one*

4.	Have you ever spoken to your GP about this problem?	**YES 1**	**NO 2**
5.	Have you spoken to your GP about this problem in the past 6 months?	**YES 1**	**NO 2**
6.	Have you ever seen a specialist for this problem? (if you are waiting for an appointment, please select 'YES')	**YES 1**	**NO 2**
7a.	Have you had a diagnosis for this problem from a doctor?	**YES 1**	**NO 2**
7b.	If so, what was the diagnosis? …………………………………………...............		

8a. Have you taken medication prescribed for this problem by a doctor in the past 6 months? **YES** [1] **NO** [2]

8b. If so, please tell us how you take them (*tick only one box*)

On a regular basis ☐ [1]

Occasionally ☐ [2]

9. Have you seen any of the following practitioners for this problem in the past 6 months?

Please tick ✓ all that apply

Acupuncturist	☐ [a]
Osteopath or chiropractor	☐ [b]
Homeopath	☐ [c]
Reflexologist	☐ [d]
Chinese medicine herbalist	☐ [e]
Herbalist (*not* Chinese medicine)	☐ [f]
Massage therapist (e.g., shiatsu, aromatherapy)	☐ [g]
Hypnotherapist	☐ [h]
Counsellor or psychotherapist	☐ [i]
Spiritual healer	☐ [j]

Another not listed above
(please say what type of therapy you received)[k]: ……………………………..……….

10. We are particularly interested in the things that you do for yourself.

Please say if you have done any of the following in the past 6 months:

	**YES, I have done this**	
	For this health problem	For a different reason
Taken over the counter medicines (tablets or creams bought without a doctors prescription, e.g., paracetamol, arnica)	☐ 2	☐ 3
Taken vitamins or supplements	☐ 2	☐ 3
Used heat (e.g., a bath or hot water bottle)	☐ 2	☐ 3
Used cold (e.g, an ice pack)	☐ 2	☐ 3
Rubbed or massaged yourself	☐ 2	☐ 3
Followed a special diet	☐ 2	☐ 3
Tried to think more positively	☐ 2	☐ 3
Spent time alone	☐ 2	☐ 3
Broken up tasks into smaller, manageable chunks (paced)	☐ 2	☐ 3
Rested much of the day	☐ 2	☐ 3
Used meditation or relaxation	☐ 2	☐ 3
Exercised (e.g., walking, swimming, exercise classes, team sport)	☐ 2	☐ 3
Contacted a friend or family member for support	☐ 2	☐ 3
Prayed	☐ 2	☐ 3
Went to a support group or social group	☐ 2	☐ 3
Cut out activities	☐ 2	☐ 3
Drank alcohol	☐ 2	☐ 3
Took part in hobbies	☐ 2	☐ 3
Went to bed early or slept in late	☐ 2	☐ 3
Other (please specify what: …………………………	☐ 2	☐ 3

11. Which of the following have you <u>used in the last 6 months</u> as a **source of information** for help with your health problem? Please tick ✓ all that apply.

Friend	☐ a	Web sites	☐ h
Family member	☐ b	Internet chat rooms/forums	☐ i
Doctor (GP)	☐ c	Newspaper or magazine	☐ j
Nurse	☐ d	Television programme	☐ k
Therapist (such as physiotherapist, psychologist)	☐ e	Support group	☐ l
Complementary therapist (such as homeopath, osteopath)	☐ f	Other (please say what):	☐ m
Pharmacist	☐ g	……………………………………………	

12. Which of the following do you <u>trust</u> as a source of information (whether or not you have used it) for help with your health problem? Please tick ✓ all that apply.

		**Yes** I trust this as a source of information
a)	Friend	☐ 1
b)	Family member	☐ 1
c)	Doctor (GP)	☐ 1
d)	Nurse	☐ 1
e)	Therapist (such as physiotherapist, psychologist)	☐ 1
f)	Complementary therapist (such as homeopath, osteopath)	☐ 1
g)	Pharmacist	☐ 1
h)	Web sites	☐ 1
i)	Internet chat rooms / online forums	☐ 1
j)	Newspaper or magazine	☐ 1
k)	Television programme	☐ 1
l)	Support group	☐ 1
m)	Other (please say what): …………………………………………………	

13. Is there anything else you think might help your health problem that you are not currently using?

Please tell us more (for example, you could tell us about why you think it might help, where you heard about it, whether you plan on trying it in future, why you aren't currently using it):

14. Is there anything you feel your GP could do to help with this health problem that is not already being done?

15. In general, would you say your health is: (circle one only)

- Excellent 1
- Very good 2
- Good 3
- Fair 4
- Poor 5

16. Are you: Male ☐ [1] Female ☐ [2]

17. Please tell us your age at your last birthday: …………… years

18. At what age did you leave school? …………… years

19. Please indicate the ethnic group to which you feel you belong (tick only one):

White:	British	☐ [1]
	Any other White background (please describe)	………………………
Mixed:	White and Black Caribbean	☐ [2]
	White and Black African	☐ [3]
	White and Asian	☐ [4]
	Any other Mixed background (please describe)[5]	………………………
Asian or Asian British:	Indian	☐ [6]
	Pakistani	☐ [7]
	Bangladeshi	☐ [8]
	Any other Asian background	☐ [9]
Black or Black British:	Caribbean	☐ [10]
	African	☐ [11]
	Any other Black background	☐ [12]
Chinese or other ethnic group:	Chinese	☐ [13]
	Any other (Please describe)[14]	………………………

20. What are your living arrangements? Please tick only one

Own your own home outright	☐ [1]	Rent from a private landlord	☐ [5]
Own your own home with a mortgage	☐ [2]	Rent from a council / housing association	☐ [6]
Live in someone else's home	☐ [3]	Live in a nursing home	☐ [7]
Live in a retirement home / sheltered accommodation	☐ [4]	Other (please describe) [8]	…………………….

21. Do you currently live: Alone ☐ [1] With another person / other people ☐ [2]

22. Do you have any other comments?

☺ You have reached the end of the questionnaire. Please now:

☑ Check that you have filled in the QUESTIONNAIRE and REPLY SLIP

☑ Return both to us using the FREEPOST envelope provided

If you have any questions or queries please contact the researcher,
Fiona MacKichan, on 01392 262913

Thank you for taking part in our study.
This research would not be possible without your help!
